# Reproductive Characteristics of Hermaphrodites in a Trioecious Marine Mussel: Evidence of a Mixed Mating Strategy

**DOI:** 10.3390/biology15201897

**Published:** 2026-10-21

**Authors:** Pablo A. Oyarzún, Gonzalo Ruiz-Tagle, Constanza Bustos, Javiera Jerez, José Garcés-Vargas, Juan A. Valdés, José J. Nuñez, Jorge E. Toro

**Affiliations:** 1 Centro de Investigación Marina Quintay (CIMARQ), Universidad Andres Bello, Quintay 2480000, Chile; gruiztagle96@gmail.com (G.R.-T.); cb.jones.1804@gmail.com (C.B.); mjjerezmorales@gmail.com (J.J.); jvaldes@unab.cl (J.A.V.); 2 Programa de Doctorado en Medicina de la Conservación, Facultad de Ciencias de la Vida, Universidad Andrés Bello, Santiago 8320000, Chile; 3 Instituto de Ciencias Marinas y Limnológicas, Universidad Austral de Chile, Avenida Rector Eduardo Morales 23, Valdivia 5090000, Chile; josegarces@uach.cl (J.G.-V.); jjnunezn@gmail.com (J.J.N.); 4 Interdisciplinary Center for Aquaculture Research—Applied Research (INCAR2), Concepción 4030000, Chile; 5 Departamento de Acuicultura y Recursos Agroalimentarios, Universidad de Los Lagos, Av. Fuchslocher 1305, Osorno 5290000, Chile; jorge.toroyagui@ulagos.cl

**Keywords:** gametogenesis, reproductive behaviors, molluscs, sexual systems, spawning, self-fertilization

## Abstract

Trioecy—the coexistence of males, females, and hermaphrodites within the same population—is one of the rarest sexual systems described in animals. The marine mussel *Semimytilus patagonicus* is among the few known examples, with hermaphrodites accounting for 95% of individuals. However, the reproductive biology of this dominant phenotype remains poorly understood. In the present study, more than 2500 individuals from natural populations were analyzed to assess sexual maturity, gametogenesis, spawning dynamics, and self-fertilization in hermaphroditic *S. patagonicus*. The results revealed exceptionally early sexual maturity (L_50_ = 6.49 mm), with the age at L_50_ estimated at approximately 68 days based on the fitted model, placing this species among the earliest-maturing bivalves reported to date. Gametogenic development exhibited a seasonal pattern characterized by a high proportion of germinal tissue throughout the study period. Furthermore, variation in male and female gametogenic tissues was synchronized. With respect to gamete release, spawning displayed a protandric pattern, in which the first spawning event was dominated by the release of male gametes (53%), whereas the second was dominated by the release of female gametes (83%), suggesting a mechanism that promotes outcrossing. In addition, fertilization experiments demonstrated that self-fertilization produced normal embryos (~86%) and larval sizes (trochophore: $\bar{x}$ = 69.4 µm; veliger: $\bar{x}$ = 118.6 µm) comparable to those obtained through cross-fertilization, indicating a functional capacity for self-fertilization without evidence of inbreeding depression during early developmental stages. This combination of reproductive traits in *S. patagonicus* is consistent with a mixed mating strategy that balances the advantages of self-fertilization with mechanisms that promote outcrossing. Such a life-history configuration may be particularly adaptive in the dynamic and heterogeneous intertidal environment inhabited by this species. We discuss the implications of these findings for understanding the reproductive dynamics of a trioecious species and their broader significance for the evolution of sexual systems in Animalia.

## Simple Summary

Hermaphroditic marine mussels are relatively rare, and little is known about their reproductive strategies and biology. This study investigated the reproductive biology of the marine mussel *Semimytilus patagonicus* along the Chilean coast, focusing on the size at sexual maturity, seasonal reproductive patterns, the release of reproductive cells, and the ability to reproduce through self-fertilization. By examining more than 2500 mussels and conducting laboratory experiments, we found that this species reaches sexual maturity at a very small body size. Its reproductive activity followed a seasonal pattern, with two main periods of reproductive cell release. During the first period, most individuals released male reproductive cells, whereas the second was dominated by the release of female reproductive cells. Laboratory experiments also showed that self-fertilization produced embryos with normal development and larvae similar in size to those produced through fertilization between two individuals. These findings suggest that this mussel can employ both reproductive strategies, potentially maintaining reproductive capacity when mates are scarce while also allowing outcrossing when mates are available.

## 1. Introduction

The various mating strategies and reproductive behaviors employed by species play a fundamental role in shaping population dynamics, genetic variation, and evolutionary processes [1]. Sexual systems are fundamental biological traits of the species, where hermaphroditism and dioecy (also known as gonochorism) are the most common in nature [2,3]. Hermaphroditism refers to the presence of both male and female reproductive structures within a single individual, allowing for self-fertilization or cross-fertilization with another hermaphrodite. Dioecy, on the other hand, refers to the separation of sexes into distinct female and male individuals, necessitating cross-fertilization for reproduction [1,4]. Furthermore, several intermediate sexual systems have been identified in animal populations, including androdioecy, which involves the coexistence of males and hermaphrodites; gynodioecy, characterized by the presence of females and hermaphrodites; and trioecy, which involves the coexistence of males, females, and hermaphrodites. Among these systems, trioecy is the rarest and least understood in both animals and plants [3]. The scarce records of species displaying this sexual system have hindered the in-depth exploration of certain aspects of their reproductive biology. In Animalia, trioecy has been reported in the nematodes *Auanema freiburgensis* and *A. rhodensis* [5,6,7], the green hydra *Hydra viridissima* [8], sea anemones *Bartholomea annulata* [9], *Aiptasia diaphana* [10], urchin *Toxopneustes roseus* [11] and the marine mussel *Semimytilus algosus* [3].

*Semimytilus patagonicus* (Hanley, 1843), currently recognized as the valid name for the species formerly referred to as *Semimytilus algosus* (Gould, 1850) (principle of priority), is a bivalve species belonging to the family Mytilidae that inhabits the intertidal zone along the Pacific coast of South America. Its distribution extends from Ecuador to Maicolpué, Chile [3]. This species forms dense intertidal beds, particularly during the summer season, covering extensive rocky surfaces in multiple layers. Simultaneous hermaphrodites dominate its populations (~95%), exhibiting both male and female gonadal tissues concurrently. Nevertheless, unisexual individuals are also present, comprising approximately 4% females and 1% males [3]. Although the functional role of these sexual phenotypes within population dynamics remains unknown, the high prevalence of hermaphroditism suggests that it constitutes the predominant reproductive strategy of the species. In this context, several questions arise: What is the reproductive strategy of a mussel with simultaneous hermaphroditism coexisting with male and female individuals within the same population? Furthermore, does *S. patagonicus* possess mechanisms that reduce or prevent the effects of inbreeding depression?

Mussels of the family Mytilidae exhibit relatively conserved reproductive characteristics. Most species are dioecious and display annual gametogenic cycles [3,12,13]. Fertilization is external and occurs following the release of gametes into the marine environment [14,15]. Subsequently, they undergo a planktonic larval phase during which larvae remain in the water column for several weeks before settling and attaching to a suitable substrate [15]. These reproductive traits constitute key adaptations that promote dispersal and population persistence in marine environments. However, *S. patagonicus* represents a notable exception within the family, as it exhibits hermaphroditism in an otherwise predominantly dioecious lineage.

The aim of this study was to characterize key aspects of the reproductive biology of *Semimytilus patagonicus*, including sexual maturity, gametogenic development, and spawning strategies.

## 2. Materials and Methods

### 2.1. Estimation of Size and Age at First Sexual Maturity

A total of 2486 mussels were collected from three locations along the Chilean coast to determine the size at first sexual maturity of the species. The specimens were collected from Algarrobo (33°35′ S; *n* = 1254) on 15 December 2023, Calfuco (39°78′ S; *n* = 555) on 10 January 2024, and Coquimbo (30°07′ S; *n* = 677) on 15 January 2024. All sampling was conducted during the spring–summer period, corresponding to peak gonadal development. For the purpose of estimating the size at first sexual maturity at the species level, data from the three locations were pooled for the analysis. All specimens were sexed and measured for total shell length (±0.1 mm). Sexual maturity was assessed through microscopic examination of the gonads, based on the presence or absence of germ cells, following the methodology described by Oyarzún et al. [3].

**Figure 1:**
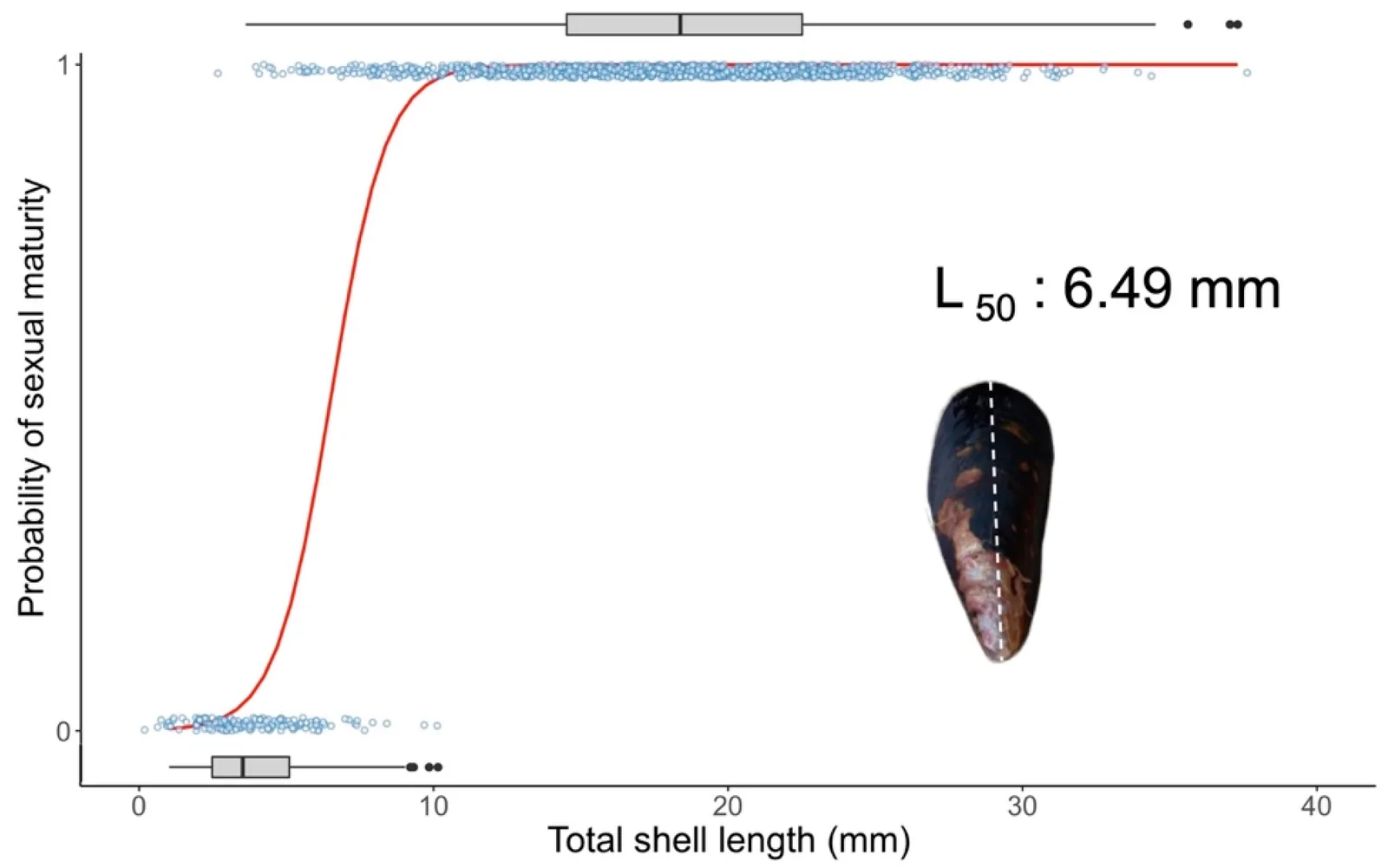
Logistic model describing the probability of sexual maturity in the mussel *Semimytilus patagonicus* as a function of total shell length (mm). Blue circles represent individual observations classified as immature (0) or mature (1), Boxplots summarize the distribution of shell lengths for immature and mature individuals.

For the analysis, a Generalized Linear Model (GLM) with a binomial distribution was employed to fit the logistic curve [16], following the equation: *p* = exp(*b*_0_ + *b*_1_ × LT)/[1 + exp(*b*_0_ + *b*_1_ × LT)], where *p* represents the probability of an individual reaching sexual maturity at a given total shell length (LT; see Figure 1); *b*_0_ and *b*_1_ are the parameters of the logistic curve. Consequently, size at 50% sexual maturity (L_50_ = −*b*_0_/*b*_1_) was estimated. The ‘tidyverse’ package v.2.0.0 [17], implemented in R project v.4.5.1 [18], was used for these analyses.

We then estimated the age at which *S. patagonicus* reaches the minimum size at sexual maturity (L_50_). To determine the age at sexual maturity, we individually marked 219 specimens collected from Algarrobo. The specimens were maintained in 5000 L tanks under a constant flow of seawater and natural photoperiod conditions. From October 2022 to July 2023, animal length was measured every 50 days to collect growth data, each measurement corresponding to one growth check. These data were fitted to the von Bertalanffy growth model, following the methodology outlined by Ogle & Isermann [19]. This fitting was carried out using the following equation: L_t_ = L_∞_[1 − exp(−K(t − t_0_))], where Lt represents the expected average length at time t, L_∞_ signifies the asymptotic length, K quantifies the exponential rate at which L_t_ approaches L_∞_, and t_0_ denotes the theoretical age at which L_t_ equals zero. Once these parameters were derived, the age at which L_50_ is attained was estimated (Figure S1). To conduct this analysis, the ‘FSA’ package [20] and ‘nlstools’ package [21] were employed, both implemented within the R project v.4.5.1 [18].

### 2.2. Histological Procedure and Gametogenic Analysis

Adult mussels (30.1 to 40.3 mm, *n* = 375 individuals) were collected from the rocky intertidal zone of Calfuco, Chile (39°78′ S), with 30–32 individuals sampled monthly over the course of one year (November 2023–October 2024). To ensure sample integrity, the animals were kept alive until the moment of dissection. A scalpel was used to excise the gonad from each specimen, which was subsequently fixed in Davidson’s solution for 48 h. Histological processing of the tissues, including dehydration and paraffin embedding, was carried out according to the methodology described by Oyarzún et al. [3,13]. Cross-sections of the gonadal lobe, with a thickness of 7 μm, were cut from each sample. The histological sections were stained with hematoxylin and eosin.

Microscopic observations of histological sections were conducted to describe the gametogenic development of *Semimytilus patagonicus*. The gamete volume fraction (GVF), representing the proportion of germinal tissue in the gonads, was estimated using the methodology described by Toro et al. [22]. Digital photographs of male and female histological sections were taken using a microscope-mounted camera, ensuring that the entire histological section was covered by capturing all the images necessary to encompass the whole section. The images were subsequently analyzed using ImageJ software v.1.52q (https://imagej.net/ij/download.html, accessed on 13 October 2026), to calculate the percentage of germinal (follicular) and interfollicular (connective) tissue. Higher GVF values indicate a greater proportion of germinal tissue within the gonads, reflecting a higher abundance of reproductive cells.

To evaluate temporal variation in the proportion of germinal tissue (%) in mussels, a Generalized Estimating Equations (GEE) approach was adopted as the primary analytical framework. This method does not require the assumption of normally distributed residuals and is well suited for analyzing bounded proportion data.

GEE models with a Binomial family and logit link function were fitted separately for each gonadal fraction (female and male), with sampling month as the explanatory variable (12 levels: November 2023–October 2024, with November 2023 as reference category) and an exchangeable correlation structure to account for the non-independence of repeated measurements within the same hermaphroditic individual. Prior to analysis, proportions were transformed following the Smithson and Verkuilen [23] correction—(y·(n − 1) + 0.5)/n—to avoid boundary values. Model fit was evaluated using the Quasi-likelihood under Independence Model Criterion (QIC). To directly assess whether male and female gonadal fractions co-varied synchronously at the individual level, Wilcoxon signed-rank paired tests were applied for each sampling month, treating both fractions of the same individual as paired observations. Statistical significance was set at α = 0.05. Synchronous decline in both gonadal fractions is interpreted as evidence consistent with a spawning event. All analyses were conducted in R Project v.4.5.1 [18].

### 2.3. Spawning

To conduct the spawning experiments, adult mussels collected from Algarrobo, Chile, were used during their peak gonadal maturity period (spring–summer). Approximately 100 adult mussels were used as broodstock for the spawning events. The individuals were subjected to a 10-day conditioning period in 250 L tanks filled with filtered seawater and were fed ad libitum with the microalga *Isochrysis galbana* throughout the conditioning period.

Spawning was induced by thermal shock, consisting of alternating water temperatures between 12 °C and 18 °C at 30 min intervals. The temperature cycles were repeated until gamete release was observed. The order of spawning (female and/or male) was recorded for each individual, and spawning events were classified according to the first and second gamete release events.

To evaluate the temporal pattern of gamete release during spawning, 30 adult mussels were monitored during two consecutive spawning events. For each spawning event, the type of gamete released was recorded as male only (♂), female only (♀), or simultaneous male–female release (♀♂) when both gonadal components released gametes during the same spawning event.

For the first spawning event, the three possible release categories were considered equally probable under the biological null hypothesis. Before spawning, both gonadal components were considered to contain gametes, and there was no a priori biological basis to assume preferential release of either the male or female component or simultaneous release of both. Therefore, a chi-square goodness-of-fit test was used to compare the observed frequencies with an expected uniform distribution (33.3% for each category; expected frequency = 10 individuals per category; *n* = 30; α = 0.05).

The second spawning event was interpreted differently because it occurred after an initial gamete-release event. The first spawning event could result in either partial or complete depletion of one or both gonadal components. Consequently, the gamete composition available for release during the second event was expected to be constrained by the outcome of the preceding spawning event. Thus, the second event was not considered an independent replicate of the first event, and no uniform expected distribution among ♂, ♀♂, and ♀ categories was assumed. Because individual transition records linking the first and second spawning events were not retained, a paired statistical analysis of individual spawning sequences could not be performed. Therefore, the second event was summarized descriptively using the observed frequencies and percentages of male and female gamete release, and its distribution was interpreted in the context of the proposed sequential depletion of gonadal components.

### 2.4. Self-Fertilization Analysis

To assess self-fertilization in *Semimytilus patagonicus*, two experimental treatments were established. Broodstock were randomly collected from the natural intertidal bed at Algarrobo during the summer season. The first treatment consisted of the fusion of gametes from the same individual (self-fertilization), whereas the second involved cross-fertilization between two hermaphroditic mussels. Each treatment was replicated using 10 experimental units, each with a volume of 1 L. For the cross-fertilization treatment, each experimental unit consisted of an independent pair of mussels. Broodstock conditioning and spawning procedures were performed as previously described in Section 2.3.

Spermatozoa and oocytes were collected individually and maintained in sterilized containers until fertilization. Fertilization was conducted using a sperm-to-egg ratio of 100:1, following the methodology described by Toro et al. [14]. Germ cell concentrations were determined using a hemocytometer. Fertilization trials were carried out in each experimental unit, which was maintained at 14 ± 0.5 °C.

At 120 min post-fertilization, 100 embryos were randomly selected from each experimental unit and examined using an inverted microscope (Carl Zeiss Microscopy, Jena, Germany) and a Neubauer chamber (Paul Marienfeld GmbH & Co. KG, Lauda-Königshofen, Germany). Embryos were classified as normal or abnormal (deformed), and the results were expressed as percentages for each treatment. At 24 h post-fertilization, larvae were fed 15,000 cells mL^−1^ of the microalga *Isochrysis galbana*. At 30 h post-fertilization, the size of 30 randomly selected trochophore larvae was measured from each experimental unit. Similarly, at 72 h post-fertilization, the size of 30 randomly selected veliger larvae was measured from each experimental unit. Larval size measurements were used to calculate the mean larval size for each experimental unit at each developmental stage. Each experimental unit was considered an independent replicate for statistical analyses.

Statistical analyses were performed using generalized linear models (GLMs). The proportion of normal and abnormal embryos was analyzed using a GLM with a quasi-binomial error distribution and a logit link function to account for overdispersion. Fertilization treatment (self- vs. cross-fertilization) was included as the explanatory variable, while the numbers of normal and abnormal embryos recorded for each experimental unit were used as the response. Each experimental unit was considered an independent replicate (*n* = 10 per treatment).

Student’s *t*-tests were used to compare mean larval size (µm) between self- and cross-fertilization treatments. Each experimental unit was considered an independent replicate. Differences between treatments were considered significant at α = 0.05.

## 3. Results

### 3.1. Age and Size at First Sexual Maturity

A total of 2486 specimens of *S. patagonicus* were examined, with a total length ranging from 1 to 37.29 mm. The relationship between the proportion of mature individuals and total length was fitted using a logistic model, whose estimated parameters were *b_0_* = −6.91 (intercept) and b1 = 1.06 (slope). From these parameters, the following equation was obtained: *p* = exp(−6.91 + 1.06 × LT)/[1 + exp(−6.91 + 1.06 × LT)]. Where ‘*p*’ corresponds to the probability of sexual maturity and ‘LT’ to the total length of the specimen.

The model estimated a length at first sexual maturity (L_50_) of 6.49 mm, with a 95% confidence interval between 6.22 and 6.77 mm. The proportion of mature individuals increased progressively with size, reaching 100% at 10.81 mm in length.

To estimate the time required to reach the minimum size at sexual maturity, growth data were obtained from 219 *S. patagonicus* individuals with initial total shell lengths ranging from 7.7 to 30.9 mm. Growth was described using the von Bertalanffy growth model, with estimated parameters of L∞ =27.68 mm (95% CI: 27.02–28.47), K = 0.3 growth check^1^ (95% CI: 0.26–0.34), and t_0_ = −1.3 growth check (95% CI: −1.61 to −0.98) (Figure S1).

The model showed a coefficient of determination (R^2^) of 0.716, indicating that approximately 71.6% of the variability in mussel total shell length was explained by the fitted growth curve. Backward extrapolation of the von Bertalanffy model indicated that individuals would have measured the minimum size at sexual maturity (L_50_), approximately 68 days before the first growth check (95% bootstrap CI: 58–81 days), corresponding to an age of −0.36 growth checks. At this age, the predicted total shell length was 6.49 mm with a 95% bootstrap CI of 5.1–7.9 mm.

### 3.2. Gametogenic Development

**Figure 2:**
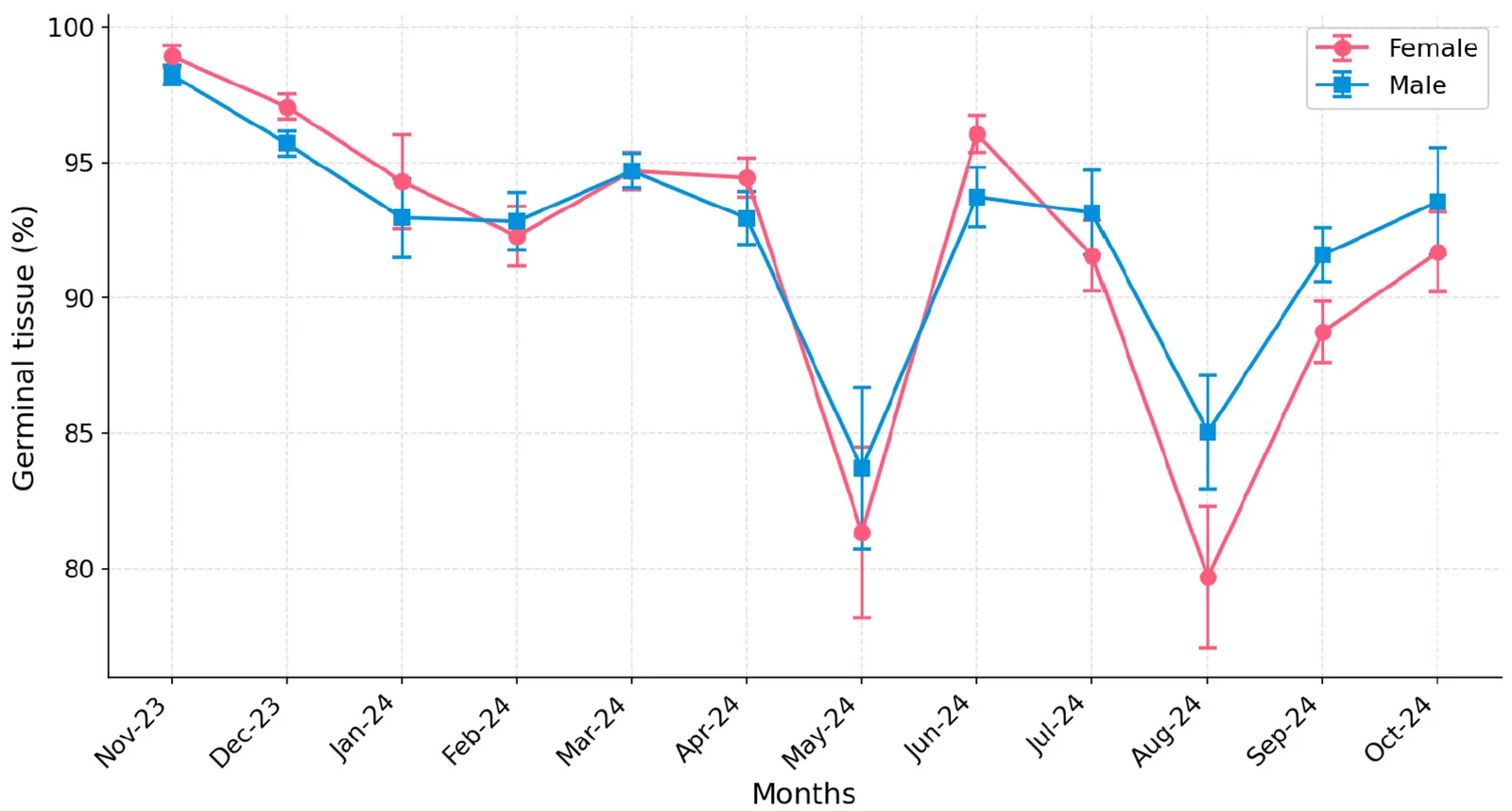
Gamete volume fraction (GVF) of gonadal tissue in simultaneous hermaphrodite mussels of the species *Semimytilus patagonicus* from the Calfuco population, Chile, evaluated between November 2023 and October 2024. The red line corresponds to the female gonadal tissue, while the blue line represents the male gonadal fraction. Error bars represent the standard error of the mean (SE).

The proportion of germinal tissue exhibited marked temporal variation throughout the sampling period in both sexes (Figure 2). Overall, values remained high (>90%) from late spring to early autumn (November 2023–April 2024), followed by pronounced declines during the autumn–winter period (May–August 2024), and a partial recovery toward the end of the sampling period (September–October 2024).

In females, germinal tissue values ranged from a maximum of 98.92 ± 0.39% in November 2023 to a minimum of 79.68 ± 4.72% in August 2024. In males, values ranged from 98.22 ± 0.37% in November 2023 to 83.69 ± 2.98% in May 2024. GEE models with a Binomial-logit link and exchangeable correlation structure confirmed significant temporal variation in germinal tissue proportion across sampling months in both the female fraction (QIC = 2.598 vs. null QIC = 6.635) and the male fraction (QIC = 2.890 vs. null QIC = 4.950), with all months differing significantly from the November 2023 baseline in both fractions (all *p* ≤ 0.005).

Two episodes of synchronous and significant declines in germinal tissue were identified in both sexes, which were interpreted as evidence of spawning events in the mussels. The first occurred in May 2024, when a sharp and simultaneous decrease in germinal tissue was observed in females (81.32 ± 3.16%, Δ = −13.1% relative to April 2024; β = −2.873, OR = 0.057, 95% CI [0.029, 0.112], *p* < 0.001) and males (83.69 ± 2.98%, Δ = −9.2% relative to April 2024; β = −2.273, OR = 0.103, 95% CI [0.061, 0.175], *p* < 0.001). The paired within-individual analysis confirmed that both gonadal fractions declined synchronously at the individual level during this month (Wilcoxon signed-rank test: W = 19.0, *p* = 0.432), with no significant difference between fractions, indicating a coordinated reproductive event during late autumn.

The second spawning event occurred in August 2024, when a second synchronous decline was observed, with females reaching 79.68 ± 4.72% (Δ = −11.9% relative to June 2024; β = −2.977, OR = 0.051, 95% CI [0.024, 0.110], *p* < 0.001) and males 85.04 ± 4.50% (Δ = −8.1% relative to July 2024; β = −2.172, OR = 0.114, 95% CI [0.056, 0.234], *p* < 0.001). Both fractions again declined synchronously at the individual level (Wilcoxon: W = 7.0, *p* = 0.297), with no significant within-individual difference between sexes. A significant difference between fractions was detected only in June 2024 (W = 2.0, *p* = 0.006), where the female fraction (96.04 ± 0.69%) had already recovered while the male fraction (93.73 ± 1.10%) had not yet reached equivalent levels, suggesting sex-specific differences in post-spawning gonadal recovery rates. The recovery observed in both sexes during September–October 2024 is consistent with post-spawning gonadal reconstitution (Figure 2).

### 3.3. Spawning Analysis

**Figure 3:**
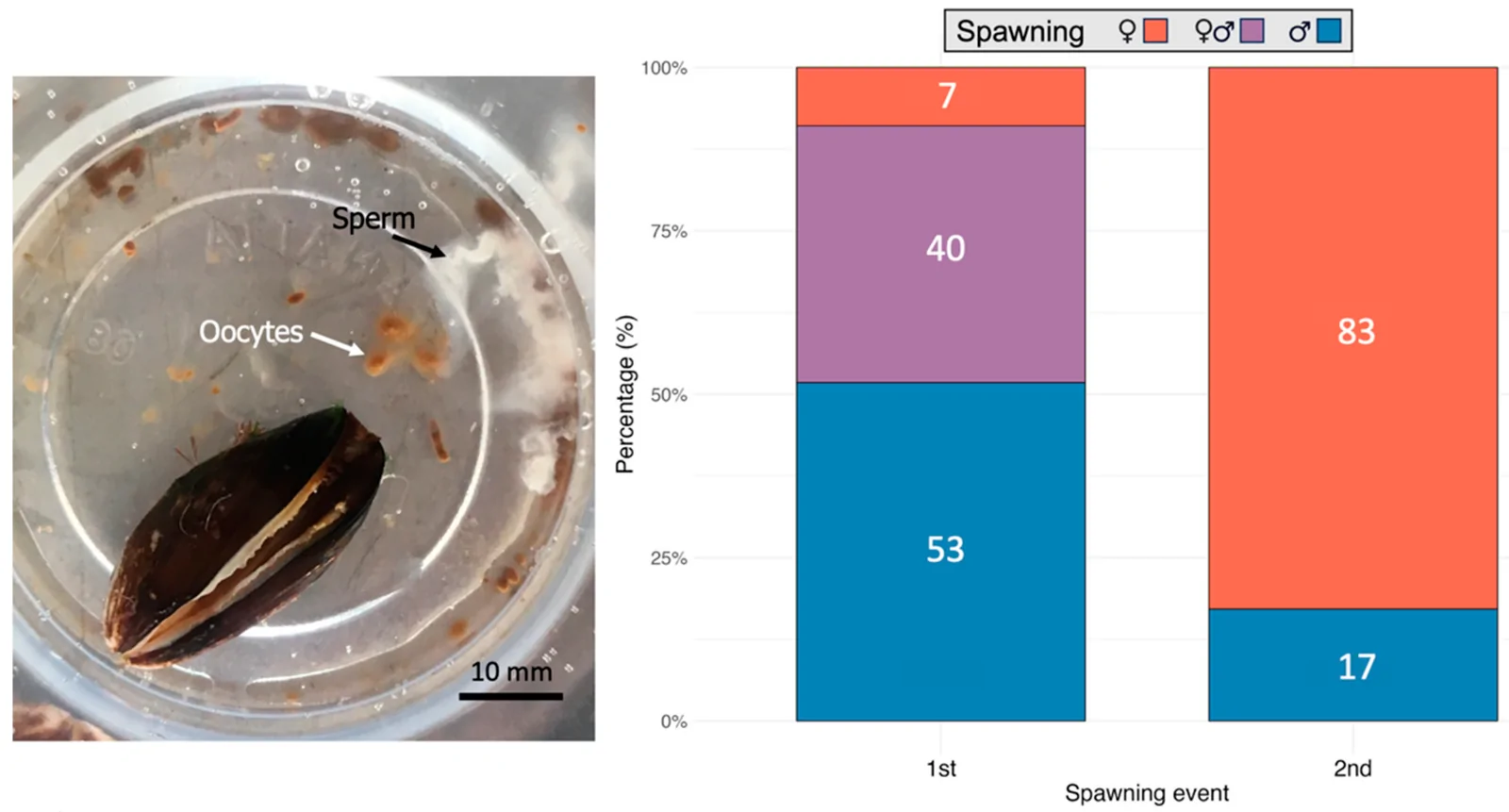
Spawning of hermaphroditic individuals of *Semimytilus patagonicus* during two reproductive events. **Left** panel: hermaphroditic individual simultaneously releasing oocytes and sperm during spawning. Arrows indicate the presence of oocytes and sperm in the surrounding water. **Right** panel: percentage distribution of individuals spawning as male, hermaphrodite, or female during the first and second spawning events. Numbers within the bars indicate the observed percentages for each sexual category. Orange indicates individuals spawning exclusively as females, blue indicates individuals spawning exclusively as males, and purple indicates hermaphroditic individuals simultaneously releasing both oocytes and spermatozoa.

During the first spawning event, male-only gamete release was observed in 16 of the 30 individuals (53.3%), simultaneous male–female release in 12 individuals (40.0%), and female-only release in 2 individuals (6.7%) (Figure 3). The observed distribution differed significantly from the expected uniform distribution of 10 individuals per category (χ^2^(2) = 10.40, *p* = 0.0055). Thus, the first spawning event was characterized by a significantly higher frequency of male-only and simultaneous male–female release and a markedly lower frequency of female-only release than expected under the null hypothesis of equal probabilities.

During the second spawning event, female gamete release was observed in 25 of the 30 individuals (83.3%), whereas male gamete release was observed in 5 individuals (16.7%) (Figure 3). Unlike the first event, the second spawning event was not evaluated against a uniform distribution because gamete availability during this event was biologically constrained by the preceding spawning event. The marked predominance of female gamete release during the second event is therefore consistent with a sequential depletion process, in which the gamete type available for subsequent release depends on the extent to which the male and female gonadal components were depleted during the first spawning event.

### 3.4. Self-Fertilization

**Figure 4:**
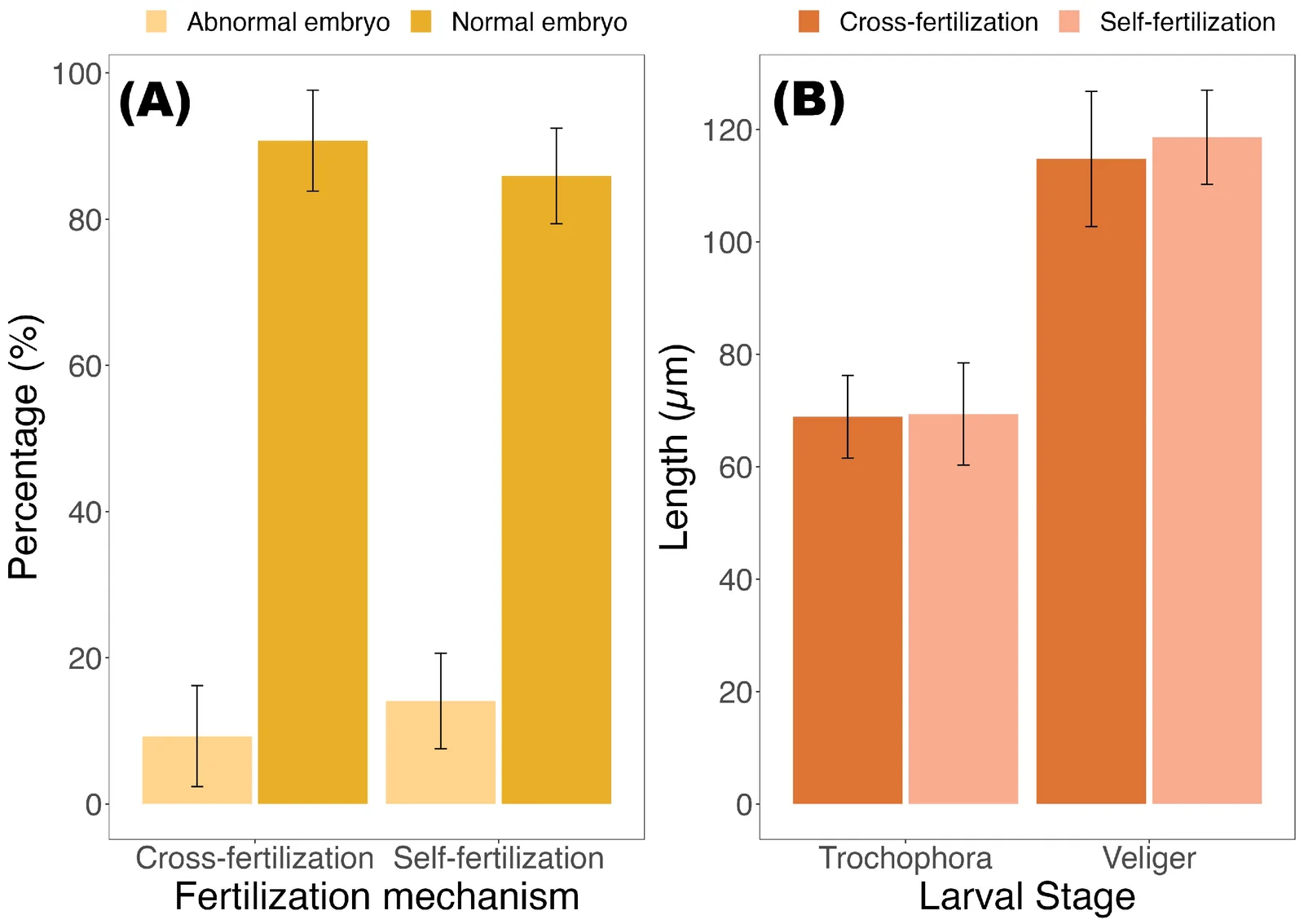
Effects of self-fertilization and cross-fertilization on embryonic and larval development in the simultaneous hermaphroditic mussel *Semimytilus patagonicus*. (**A**) Percentage of normal and abnormal embryos produced under cross-fertilization and self-fertilization treatments. (**B**) Mean larval length of trochophore and veliger larvae obtained from cross-fertilization and self-fertilization treatments. Error bars represent the standard deviation (SD).

From 20 min post-fertilization onwards, the formation of the fertilization envelope and polar bodies was observed in both treatments. Embryonic development in *Semimytilus patagonicus* did not differ significantly between self- and cross-fertilization treatments. Cross-fertilization resulted in 90.7 ± 6.9% normal embryos, compared with 85.9 ± 6.5% in the self-fertilization treatment. A generalized linear model with a binomial error distribution showed no significant effect of fertilization treatment on the proportion of normal embryos after accounting for overdispersion (F_1,18_ = 2.40, *p* = 0.139; *n* = 10 per treatment). The proportion of abnormal embryos was 9.3 ± 6.9% in the cross-fertilization treatment and 14.1 ± 6.5% in the self-fertilization treatment (Figure 4A).

Larval growth was also similar between fertilization treatments. Trochophore larvae reached mean lengths of 68.87 ± 7.35 µm and 69.37 ± 9.09 µm in the cross- and self-fertilization treatments, respectively, with no significant difference between treatments (t = −0.04, df = 18, *p* = 0.966; *n* = 10 per treatment). Similarly, veliger larvae reached mean lengths of 114.75 ± 12.03 µm in the cross-fertilization treatment and 118.60 ± 8.37 µm in the self-fertilization treatment (t = −0.26, df = 18, *p* = 0.796; *n* = 10 per treatment) (Figure 4B).

## 4. Discussion

The results obtained in this study provide a comprehensive perspective that contributes to understanding the reproductive strategy and population dynamics of a species exhibiting trioecy, a rare sexual system that remains poorly understood within the Animalia kingdom.

### 4.1. Precocious Sexual Maturity in the Intertidal Zone

The estimated size at first sexual maturity (L_50_ = 6.49 mm) and the time required to reach it (~68 days) place *S. patagonicus* among the earliest-maturing mussels reported in the literature (e.g., [24,25,26]). This reproductive precocity is consistent with life-history theory, which predicts that organisms exposed to physically variable environments or high levels of extrinsic mortality tend to invest in reproduction at an early age as an adaptive strategy to maximize reproductive success before death [27]. This pattern has been documented in intertidal organisms, where environmental stress and physical instability favor rapid life cycles and early maturation (e.g., [28]). In other mytilid species, both L_50_ values and maturation times are considerably greater. For example, the Atlantic blue mussel *Mytilus platensis* reaches L_50_ at approximately 11.8 mm [26], whereas the giant mussel *Choromytilus chorus* attains sexual maturity at 38.40 mm [29]. Similarly, the intertidal mussel *Brachidontes rodriguezii* matures at 7.05 mm in females and 8.13 mm in males [30]. Therefore, the relatively small size at sexual maturity observed in *S. patagonicus* appears to be a distinctive reproductive characteristic of this species within the Mytilidae. Although the evolutionary origin of this trait cannot be established from the present comparative observations alone, its occurrence may be associated with the species’ unusual sexual system.

From an evolutionary perspective, the early sexual maturation observed in a predominantly hermaphroditic population can be interpreted within the framework of sex-allocation theory and the size-advantage model [31,32]. In the simultaneous hermaphrodites of *S. patagonicus*, the early onset of reproduction may be driven by the relatively low energetic cost of sperm production compared with oogenesis [32]. Consequently, small individuals may achieve reproductive functionality even when their female reproductive capacity remains limited, allowing sexual maturity to be attained at a smaller size than in strictly gonochoric species. This pattern becomes particularly evident when compared with the sympatric gonochoric mytilid *Perumytilus purpuratus*, whose size at first sexual maturity has been estimated at 8–10 mm [24], a value greater than that observed for *S. patagonicus*. Given the high prevalence of hermaphrodites (~95%) reported in populations of *S. patagonicus* [3], it is plausible that early and simultaneous investment in both sexual functions may enhance reproductive opportunities by allowing individuals to contribute to reproduction through both male and female functions under the highly variable environmental conditions characteristic of rocky intertidal habitats.

### 4.2. Gametogenesis in Simultaneous Hermaphrodites

The gametogenic cycle of hermaphroditic *Semimytilus patagonicus* mussels exhibited a seasonal pattern, characterized by a high proportion of germinal tissue (>90%) from late spring to early autumn (November 2023–April 2024), followed by two synchronous declines (spawning events) during autumn (May 2024) and winter (August 2024), and a subsequent phase of gonadal recovery. Similar reproductive patterns have been reported in other mytilid species along the Chilean coast. Oyarzún et al. [12] documented spawning pulses during autumn and winter in *Mytilus chilensis*, while comparable observations have also been reported for the intertidal mussel *Perumytilus purpuratus* [13]. The synchronous decline in germinal tissue observed in both sexual functions during each spawning event indicates that male and female gametogenesis are temporally co-regulated within the same individual.

In simultaneous hermaphrodites, the co-regulation of male and female gametogenesis raises important questions regarding the endocrine and molecular mechanisms involved. In hermaphroditic gastropods and opisthobranchs, neuropeptides and steroid hormones have been shown to modulate the relative investment allocated to each sexual function [33,34]. However, it remains unknown whether equivalent regulatory mechanisms operate in hermaphroditic bivalves, particularly in species with simultaneous sexual functionality. In our study, the close temporal coupling observed between both gametogenic lineages suggests the existence of a common physiological signal—typically associated with seawater temperature or nutritional status—that may concurrently coordinate both male and female gametogenesis [13,35,36,37].

It is important to note that, although the analyses were conducted exclusively on hermaphroditic individuals, the germinal tissue volume fraction data provide a robust characterization of the reproductive cycle of the dominant sexual phenotype within the population. Future studies should incorporate unisexual males and females to determine whether these individuals share the same reproductive seasonality as hermaphrodites or exhibit divergent gametogenic strategies.

### 4.3. Sequential Gamete Release

One of the most significant findings of this study is the non-random and sequential pattern of gamete release observed in the simultaneous hermaphrodites of *S. patagonicus*. During the first spawning event, male gamete release predominated, whereas female gametes dominated during the second event (83% exclusive oocyte release). This pattern constitutes what we refer to as a protandric expression of reproductive function, whereby simultaneous hermaphrodites collectively tend to release sperm before oocytes during a reproductive season. Importantly, this pattern reflects a temporal sequence in gamete release rather than a change in sexual phenotype. The sequential release of male gametes prior to female gametes may be interpreted as a mechanism that maximizes cross-fertilization while minimizing self-fertilization [38,39]. If an individual’s sperm are released before its own oocytes become available, the likelihood of self-fertilization is reduced, as sperm can fertilize the oocytes of other individuals in the water column before the individual’s own eggs are released. The pronounced shift toward female-biased gamete release observed during the second spawning event further supports this compensatory strategy for reducing selfing [38,40].

### 4.4. Self-Fertilization in Semimytilus patagonicus

Our results demonstrated that self-fertilization in *Semimytilus patagonicus* produced embryos and larvae with developmental success comparable to that obtained through cross-fertilization. The absence of detectable inbreeding depression during the early developmental stages contrasts with observations reported for other hermaphroditic invertebrates, in which self-fertilization is associated with reduced viability, developmental abnormalities during embryogenesis, and smaller larval size, all of which are manifestations of inbreeding depression resulting from the expression of deleterious recessive alleles (e.g., [41,42,43]). To our knowledge, this study provides the first evidence of viability associated with self-fertilization in marine mussels. Nevertheless, long-term studies assessing survival and growth through to adulthood are needed to fully understand the impact of this reproductive mode on individual fitness.

Under conditions of low population density and/or restricted dispersal—scenarios that commonly occur in intertidal habitats following disturbance events—the ability to reproduce through self-fertilization may confer a reproductive advantage. This capacity has been recognized as an adaptive advantage in colonizing species and in populations subject to Allee effects [44]. However, if self-fertilization occurs frequently in natural populations, it may lead to increased homozygosity and a reduction in long-term adaptive potential [45]. Given that *Semimytilus patagonicus* has been reported as an invasive species in South Africa [46], we hypothesize that the ability to self-fertilize may have contributed to its capacity for rapid colonization and establishment in novel environments. This hypothesis is based on the potential reproductive advantage of self-fertilization under conditions of low population density or when conspecific mates are scarce, and these aspects warrant further investigation.

Taken together, the functional capacity for self-fertilization, combined with spawning behavior, suggests a mixed mating strategy in *Semimytilus patagonicus*. Although hermaphroditic mussels are capable of self-fertilization, behavioral mechanisms such as the sequential release of gametes appear to promote cross-fertilization under natural conditions. The evolutionary stability of mixed mating systems has been explained by models in which self-fertilization increases under mate limitation, whereas cross-fertilization predominates when reproductive opportunities are abundant [47,48]. In *S. patagonicus*, the predominance of hermaphroditic individuals (~95%) within intertidal beds may increase the potential for cross-fertilization by providing a high availability of potential mating partners. However, whether this population structure results in lower rates of self-fertilization remains to be determined.

### 4.5. Reproductive Ecology in the Context of Trioecy

Trioecy, the coexistence of males, females, and hermaphrodites within the same population, is the rarest sexual system found in nature [3,49]. The reproductive dynamics of trioecious species are considered complex; consequently, trioecy is generally viewed as an evolutionarily unstable sexual system that emerges as a transitional state between gonochorism and hermaphroditism [3,50].

In this study, we focused exclusively on hermaphroditic individuals, obtaining key insights into the biology of the dominant sexual phenotype. Synchronous gametogenesis, coordinated spawning of male and female gonadal tissues, and the capacity for self-fertilization suggest that hermaphroditic mussels are functionally self-sufficient from a reproductive perspective. However, it remains unclear whether unisexual individuals (males and females) share the same reproductive seasonality as hermaphrodites or, alternatively, contribute to reproduction during different periods of the year. Addressing these questions will be important for a more comprehensive understanding of the reproductive ecology of this species.

## 5. Conclusions

Understanding sexual systems provides crucial insights into the ecological and evolutionary processes shaping animal reproduction. The reproductive biology of hermaphroditic individuals of *S. patagonicus* revealed reproductive traits consistent with a mixed mating strategy, in which self-fertilization may provide reproductive assurance while cross-fertilization may confer the inherent benefits of outcrossing. This reproductive strategy may represent an adaptation to the dynamic and spatially heterogeneous intertidal environment inhabited by this species. However, as natural self-fertilization rates were not directly measured, further studies are needed to determine the relative contribution of self- and cross-fertilization in natural populations. Future research should also focus on sex allocation among the different sexual phenotypes to achieve a more comprehensive understanding of the reproductive biology of this species.

## Data Availability

All data generated or analyzed during this study are included in the published article (and its Supplementary Information Supplementary Files).

## Supplementary Materials

The following supporting information can be downloaded at: https://www.mdpi.com/article/10.3390/biology15201897/s1, Figure S1. Von Bertalanffy growth model fitted to total length (L, mm) of the mussel *Semimytilus patagonicus* as a function of growth checks (t), where ‘t’ is expressed in 50-day intervals. Black dots represent individual observations, while the red line indicates the mean length predicted by the model E[L∣t] = L∞(1 − e^−K(t −t0)^). The shaded area represents the 95% confidence intervals of the predicted mean length. The model explained 71.6% of the observed variation (R^2^ = 0.716), with an estimated asymptotic length of L∞ = 27.68 mm, growth coefficient K = 0.3 growth checks^−1^, and t_0_ = −1.3 growth checks. The estimated length at t = −0.36 growth checks (approximately 68 days before the first growth check) was approximately 6.5 mm (L_50_ = 6.49 mm), with a 95% confidence interval ranging from 5.1 to 7.9 mm.

## Abbreviations

The following abbreviations are used in this manuscript:

GLM	Generalized Linear Model
QIC	Quasi-likelihood under Independence Model Criterion
LT	Total shell length
L_∞_	Asymptotic length
L_50_	Size at 50% sexual maturity
K	von Bertalanffy growth coefficient
GVF	Gamete Volume Fraction
GEE	Generalized Estimating Equations
