# Supplementary material for: Reproductive Characteristics of Hermaphrodites in a Trioecious Marine Mussel: Evidence of a Mixed Mating Strategy
Source: Biology (Basel). 2026 Oct 21;15(20):1897. doi: 10.3390/biology15201897 (PMC13701294; doi:10.3390/biology15201897)
Supplement: Supplementary file 1 [file biology-15-01897-s001.zip › biology-4503239-supplementary.pdf]

Supplementary material

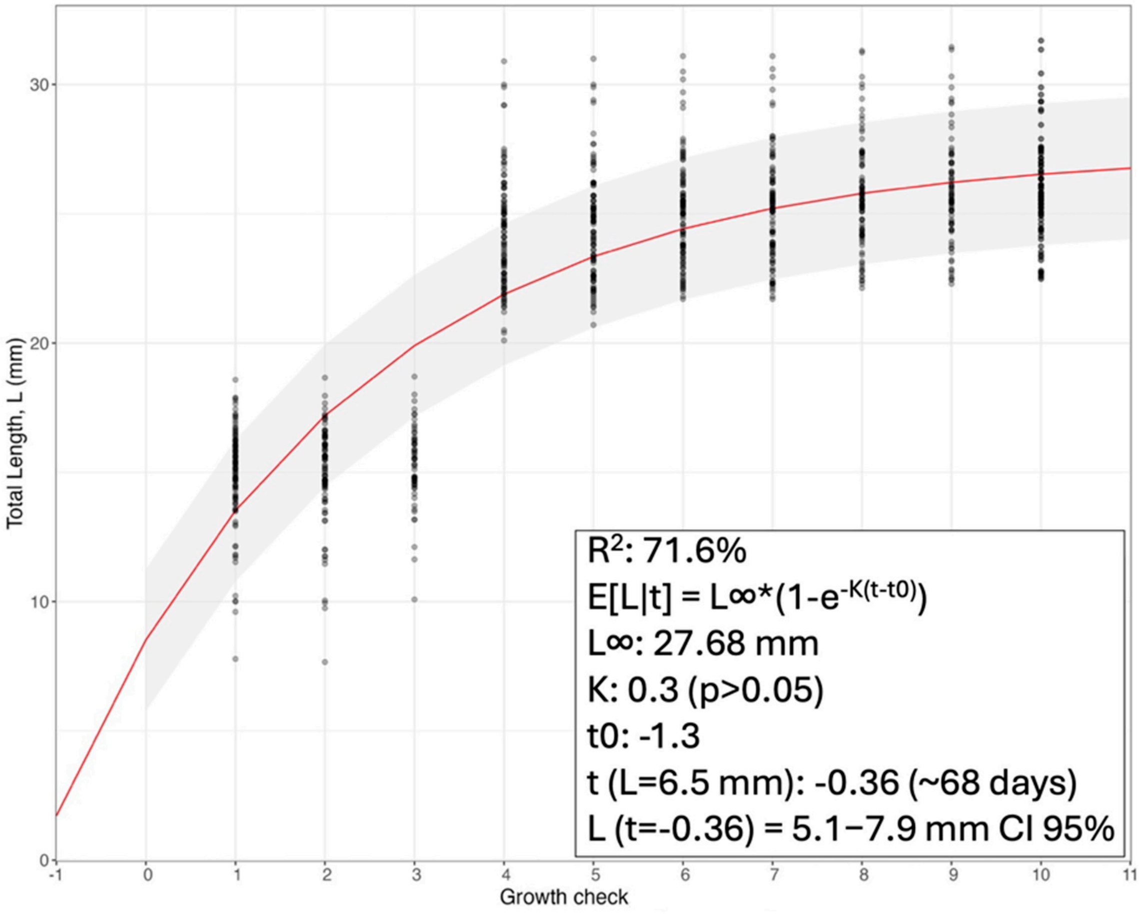

Figure S1. Von Bertalanffy growth model fitted to total length (L, mm) of the mussel *Semimytilus patagonicus* as a function of growth checks (t), where ´t´ is expressed in 50-day intervals. Black dots represent individual observations, while the red line indicates the mean length predicted by the model $E[L|t]=L\infty(1-e^{-K(t-t0)})$. The shaded area represents the 95% confidence intervals of the predicted mean length. The model explained 71.6% of the observed variation (R^2=0.716), with an estimated asymptotic length of $L\infty$=27.68 mm, growth coefficient K=0.3 growth checks^{-1}, and t_0=−1.3 growth checks. The estimated length at t=−0.36 growth checks (approximately 68 days before the first growth check) was approximately 6.5 mm (L_{50} = 6.49 mm), with a 95% confidence interval ranging from 5.1 to 7.9 mm.
